# Biological characteristics comparison of HBV rtA181T mutants with truncated or substituted HBsAg expression *in vitro* and *in vivo* model systems

**DOI:** 10.1038/srep39260

**Published:** 2016-12-15

**Authors:** Ling-Yun Zhou, En-Qiang Chen, Meng-Lan Wang, Lan-Lan Chen, Cui-Ping Liu, Fan Zeng, Hong Tang

**Affiliations:** 1 Center of Infectious Diseases, West China Hospital, Sichuan University, Chengdu 610041, China; 2 Division of Infectious Diseases, State Key Laboratory of Biotherapy and Cancer Center, West China Hospital, Sichuan University, Chengdu 610041, China

## Abstract

The hepatitis B virus(HBV) polymerase rtA181T mutation is selected during long-term antiviral therapy. As the polymerase gene completely overlaps with the envelope (S) gene, HBV rtA181T mutation also carries sW172 mutations. In this study, we investigated whether there were biological differences between rtA181T/sW172* (coding truncated HBsAg) and rtA181T/sW172L (coding substituted HBsAg) mutants. In cell experiments, a slight decline of viral replication was observed in both two mutants as compared to wild-type strains, but the levels of supernatant HBsAg and HBV DNA in rtA181T/sW172* were significantly lower than those in rtA181T/sW172L transfected cells. In animal experiments, we were amazed to find that viral replication in rtA181T/sW172* mutant increased and maintained significantly longer than that in rtA181T/sW172L mutant, while no significant difference was observed between rtA181T/sW172L and wild-type strains. Compared with wild-type strains, there were intracellular accumulations of HBsAg and HBcAg in rtA181/sW172* but none in rtA181/sW172L mutant strains. Importantly, we also found that truncated HBsAg could increase the activity of HBV core promoter, but substituted HBsAg could not. In summary, the characteristics of above two rtA181T mutants mentioned above were significantly different, and it is necessary and important for us to distinguish sW172* truncated mutation from sW172L substituted mutation.

Nucleos(t)ide analogues(NAs) have been used widely for chronic hepatitis B(CHB) treatment, which significantly improves the long-term outcomes of patients. Because all available NAs selectively target the reverse transcriptase (RT) domain of HBV DNA polymerase, mutations in HBV-RT region were naturally screened during the long term use of NAs^1^. The emergence of these mutants would inevitably lead to viral breakthrough of antiviral agent in use, and other NAs also might fail to achieve an antiviral effect because of cross-resistance phenomenon^2^,^3^,^4^,^5^.

As we known, HBV DNA has a very compact coding organization with four partially overlapping open reading frames (ORFs), including ORF P, X, S and C^6^. Among them, ORF P that encodes the RT domain of the polymerase overlaps completely with the ORF S that encodes HBV surface proteins. Hence, each mutation occurring in the RT region implies possible changes in the S region. Importantly, some of these mutations are likely to lead to a change of biological characteristics of the virus, such as the changes in replication and pathogenicity^7^.

In the past decade, lamivudine (LAM) and adefovir dipivoxil (ADV) were widely used in China. However, due to the low genetic barrier, the HBV rtA181T/V mutations are selected during the long-term treatment of LAM or ADV, especially among patients with high viral load^8^. As we known, HBV rtA181T mutant is an A→T mutation at position 181 of the P gene in the RT domain. In theory, its overlapping S gene should have two types of mutation at amino acid 172, which include rtA181T/sW172L (TGG CTC→TTA CTC) mutant coding substituted HBsAg protein and rtA181T/sW172*(TGG CTC→TGA CTC) mutant coding truncated HBsAg protein. And both two mutations are usually detected as a mixed population with wild-type HBV. So, for getting a full understanding of HBV rtA181T mutants, the biological characteristics of both sW172L and sW172* mutants should be investigated.

Recently, the HBV rtA181T/sW172* mutant has been strongly concerned, and the emergence of this mutant has reported an increased risk of hepatocellular carcinoma in LAM- or ADV-treated patients^9^,^10^. And our preliminary study also has revealed that the HBV rtA181T/sW172* mutant has a dominant secretion defect of HBsAg and viral DNA replication enhancement in liver tissue of mouse model with HBV replication^11^. However, it is worth to mention that the viral replication changes of this mutant are inconsistent in different *in vitro* cell studies. For example, the result reported by Dr.Hiromi Yatsuji showed that there was no noticeable difference in replication ability between the rtA181T/sW172* and rtA181T/sW172L^12^; while other two *in vitro* studies reported a reduced replication of rtA181T/sW172* mutant^13^,^14^. Thus, to better and fully understand the characteristics of the HBV rtA181T mutant, we designed this study to investigate the impact of different overlapping S gene mutation (sW172L and sW172*) on rtA181T mutant DNA replication and viral protein expression, not only *in vitro* in cultured HepG2 cells but also *in vivo* in mouse model.

## Results

### HBV replication and viral proteins expression in HepG2 cells *in vitro*

**Figure 1:**
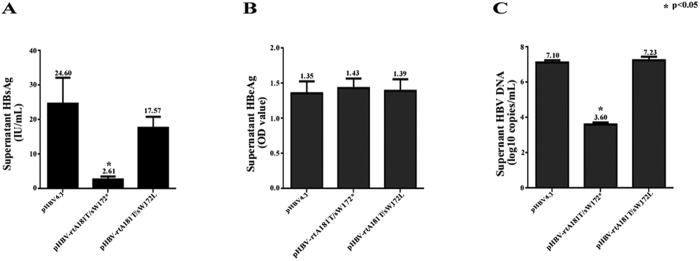
HBV proteins and DNA levels of HepG2 cell supernatant. HepG2 cells were transiently transfected with 10 μg pHBV4.1, pHBV-rtA181T/sW172* and pHBV-rtA181T/sW172L respectively. Cell supernatant was collected 3 days after plasmid transfection. (**A**) HBsAg levels in cell supernatant; (**B**) HBeAg levels in cell supernatant; (**C**) HBV DNA levels in cell supernatant. The mean and standard deviations of variables were also shown and they were obtained from three independent experiments. The *P* values were determined using Mann-whitney U test.

**Figure 2:**
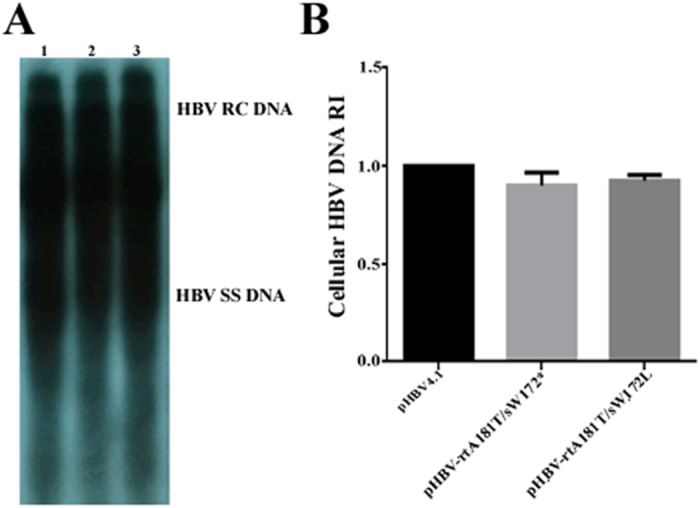
Viral replication after plasmid transient transfection in HepG2 cells. HepG2 cells were transiently transfected with 10 μg pHBV4.1, pHBV-rtA181T/sW172* and pHBV-rtA181T/sW172L respectively. Cells were collected 3 days after transfection. (**A**) HBV DNA replication intermediates of HepG2 cells were detected by Southern blotting: 1: pHBV4.1 group 2: pHBV-rtA181T/sW172* group 3: pHBV-rtA181T/sW172L group. (**B**) Quantitative analysis of HBV DNA replication intermediates. The mean and standard deviations of HBV DNA were also shown and the levels of HBV DNA replication intermediates in pHBV4.1 group 72 hours after transfection was defined as 1.

As compared to wild-type pHBV4.1 transfected cells, both the supernatant HBsAg and HBV DNA levels decreased significantly in pHBV-rtA181T/sW172* transfected cells, but not that obvious in pHBV-rtA181T/sW172L transfected cells(Fig. 1A and C). But in respect to supernatant HBeAg titre, there was no significant difference among wild-type pHBV4.1, pHBV-rtA181T/sW172* or pHBV-rtA181T/sW172L transfected cells(Fig. 1B). For the cellular HBV DNA replication intermediates, there was a small decline in both pHBV-rtA181T/sW172* and pHBV-rtA181T/sW172L transfected cells as compared to wild-type pHBV4.1 transfected cells(Fig. 2).

### The serum levels of viral proteins and HBV DNA *in vivo* mouse model

**Figure 3:**
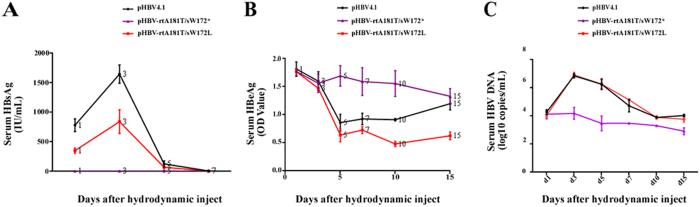
Serum levels of HBV proteins and DNA levels in mice model. Mice were injected hydrodynamically with 10 μg pHBV4.1, pHBV-rtA181T/sW172* and pHBV-rtA181T/sW172L respectively, and were sacrificed at different time points. (**A**) HBsAg levels in mice serum; (**B**) HBeAg levels in mice serum; (**C**) HBV DNA levels in mice serum. The mean and standard deviations of variables were also shown and they were obtained from three independent experiments. The *P* values were determined using Mann-whitney U test.

In wild-type pHBV4.1 injected mice, the serum levels of HBsAg could be detected since day 1 and reached a peak at day 3 after injection. The serum levels of HBsAg became lower since day 5 after injection. Similar dynamic change trend was also observed in pHBV-rtA181T/sW172L injected mice, though the levels of HBsAg were relatively lower than that in wild-type pHBV4.1 injected mice. However, the levels of HBsAg were extremely lower in pHBV-rtA181T/sW172* injected mice, and no peak was observed (Fig. 3A). In respect to serum HBeAg levels, there were no obvious differences among wild-type and two mutants injected mice either at the day 1 or day 3. Interesting, at the day 5 to day 15, serum HBeAg levels in pHBV-rtA181T/sW172* injected mice were significant higher than that in either wild-type pHBV4.1 or pHBV-rtA181T/sW172L injected mice(Fig. 3B).

The serum levels of HBV DNA in HBV-replication plasmid injected mice were also shown in Fig. 3C. The serum HBV DNA test results of the wild-type pHBV4.1 were 4.27 ± 0.16, 6.85 ± 0.15, 6.24 ± 0.35, 4.71 ± 0.44, 3.89 ± 0.05, 4.02 ± 0.08 log10 copies/mL on day 1, 3, 5, 7, 10, 15 respectively; the serum HBV DNA test results of the rtA181T/sW172* mutant strain were 4.11 ± 0.26, 4.17 ± 0.42, 3.46 ± 0.52, 3.47 ± 0.07, 3.30 ± 0.05, 2.90 ± 0.24 log10 copies/mL on day 1, 3, 5, 7, 10, 15 respectively; the serum HBV DNA test results of the rtA181T/sW172L mutant strain were 4.03 ± 0.27, 6.91 ± 0.19, 6.25 ± 0.40, 5.13 ± 0.04, 3.88 ± 0.12, 3.76 ± 0.21 log10 copies/ mL on day 1, 3, 5, 7, 10, 15 respectively There were no obvious differences in HBV DNA levels between wild-type pHBV4.1 and pHBV-rtA181T/sW172L injected mice. However, the serum levels of HBV DNA in pHBV-rtA181T/sW172* injected mice were significantly lower than that of either wild-type pHBV4.1 or pHBV-rtA181T/sW172L injected mice.

### The levels of viral replication intermediates in liver tissue *in vivo*

**Figure 4:**
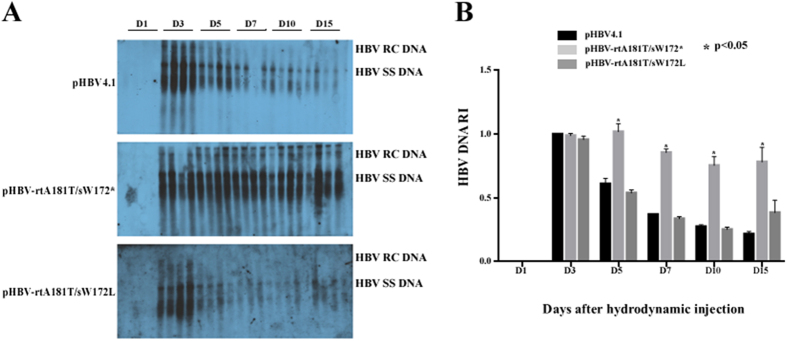
Viral replication after hydrodynamic transfection in mice model. Mice were injected hydrodynamically with 10 μg pHBV4.1, pHBV-rtA181T/sW172* and pHBV-rtA181T/sW172L respectively, and were sacrificed at different time points. (**A**) HBV DNA replication intermediates of mice livers were detected by Southern blotting. (**B)** Quantitative analysis of HBV DNA replication intermediates. The level of HBV DNA replication intermediates in pHBV4.1 group 3 days after infection was defined as 1. The mean HBV DNA levels plus standard deviation from three independent analyses were also shown, and the *P* values were determined using Mann-whitney U test.

The levels of HBV DNA replication intermediates in liver tissue of HBV-replication plasmid injected mice were shown in Fig. 4. In the liver of wild-type pHBV4.1 injected mice, HBV DNA replication intermediates were not detectable at day 1, became peaked on day 3, and decreased since day 5. And the levels of HBV DNA replication intermediates were extremely low from day 10 to day 15. Similar findings were also observed in pHBV-rtA181T/sW172L injected mice, though there seems to be weakened expression as compared to wild-type pHBV4.1. However, the levels of HBV DNA replication intermediates in pHBV-rtA181T/sW172* injected mice maintained high for 15 days, which is significantly different from that in either wild-type pHBV4.1 or pHBV-rtA181T/sW172L injected mice.

### Expression levels of HBsAg and HBcAg in liver tissue *in vivo*

**Figure 5:**
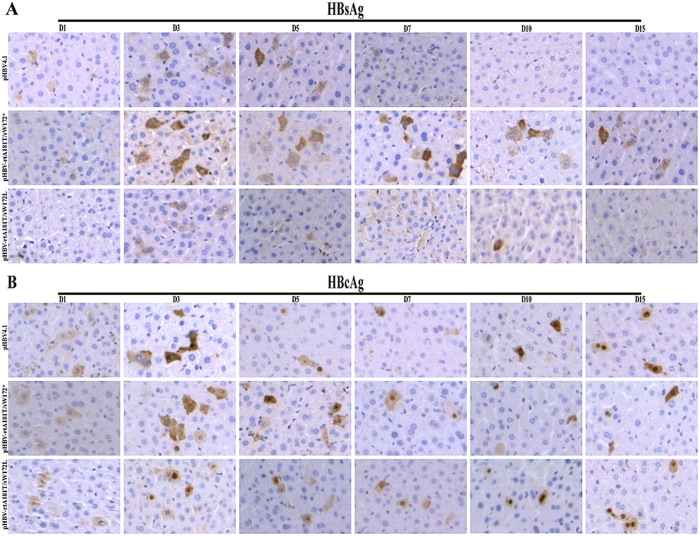
Representative images of the expression of HBsAg (**A**) and HBcAg (**B**) immunohistochemistry detection in mice liver tissue sections. Mice liver sections on day 1, 3, 5, 7, 10 and 15 after transfection were detected using specific antibody. The positive expressions were stained brown (100×original magnification).

In liver tissue of wild-type pHBV4.1 or pHBV-rtA181T/sW172L injected mice, the number of HBsAg staining cells reached its peak on day 3, reduced since day 5 and became scarce from day 10 to day 15. In contrast, the number of HBsAg staining cells in pHBV-rtA181T/sW172* injected mice was significantly increased, and the signal strength of HBsAg staining was also significantly high from day 3 to day 15 (Fig. 5A). It is worth to mention that the expression pattern of HBcAg was similar to that of HBsAg in liver tissue of wild-type or mutant HBV-replication plasmid injected mice (Fig. 5B).

### The impaction of wild-type HBsAg and mutant HBsAg on promoter regulation function

**Figure 6:**
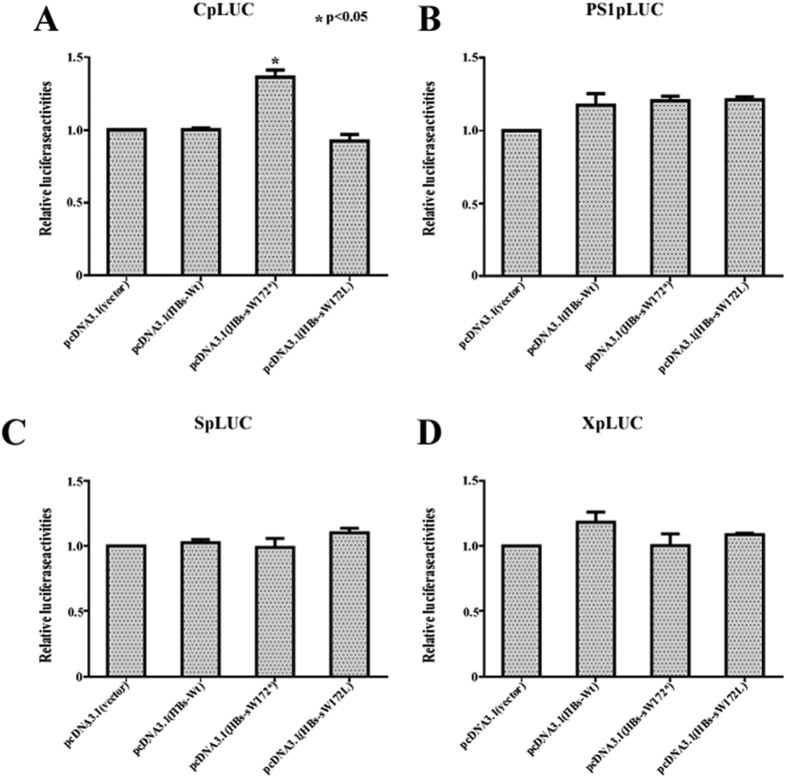
The Dual-luciferase activity of the eukaryotic expression plasmid transfected HepG2 cells. (**A**) the relative luciferase activity of CpLUC; (**B**) the relative luciferase activity of PS1pLUC; (**C**) the relative luciferase activity of SpLUC (**D**) the relative luciferase activity of XpLUC. The mean and standard deviations of relative luciferase activity were also shown and they were obtained from three independent experiments. The *P* values were determined using Mann-whitney U test.

As compared to negative control pcDNA3.1 transfected cells, the relative luciferase activity of CpLUC increased 40% in pcDNA-HBs(sW172*) transfected cells; but no obvious change was observed in either pcDNA-HBs(wt) or pcDNA-HBs (sW172L) transfected cells (Fig. 6A). It is worth to mention that there was no statistical difference of the relative luciferase activity of either PS1pLUC, SpLUC or XpLUC among pcDNA-HBs(wt), pcDNA-HBs (sW172L) and pcDNA-HBs (sW172L) tranfected cells (Fig. 6B). These results indicated that the only truncated HBsAg could enhance HBV C promoter activity, while the wild-type HBsAg and substituted HBsAg could not.

## Discussion

In this study, the impact of different mutations in the rtA181T (rtA181T/sW172* and rtA181T/sW172L) on viral DNA replication and protein expression was investigated in both *in vitro* and *in vivo* conditions. The major findings of present study were: (1) levels of HBsAg and HBV DNA in cell supernatant and blood serum in HBV rtA181T/sW172* mutant were significantly lower than in either HBV rtA181T/sW172L mutant or wild-type HBV; (2) levels of HBV DNA RI in HBV rtA181T/sW172* mutant were higher and more persistent than in HBV rtA181T/sW172L mutant and wild-type HBV; (3) significant higher levels of HBsAg and HBcAg in liver of HBV rtA181T/sW172* mutant as compare to HBV rtA181T/sW172L mutant and wild-type HBV; (4) the C promoter activity of HBV enhanced by truncated HBsAg, instead of by substitute or wild-type HBsAg. Thus, it is obvious that HBV rtA181T/sW172* and rtA181T/sW172L mutants have different biological characteristics.

It is well-known that the complete HBV virion consists of an icosahedral nucleocapsid core and an outer lipid envelope, and only the mature nucleocapsid enveloped by surface proteins can be released to serum^15^. Evidences have shown that there were three envelope proteins (including small, medium and large HBsAg) which are anchored as transmembrane proteins and could directly or indirectly affect the biological characteristics of the HBV^16^. As we mentioned above, rtA181T/sW172L (TGG CTC → TTA CTC) mutant viral strain codes substituted HBsAg protein and rtA181T/sW172*(TGG CTC → TGA CTC) mutant viral strain codes truncated HBsAg protein. Thus, we speculated that the differences in DNA replication and protein secretion between rtA181T/sW172* and rtA181T/sW172L mutant viral strains should be closely related to the different HBsAg expression.

In this study, the cell supernatant and blood serum levels of HBsAg were different between HBV rtA181T/sW172* and rtA181T/sW172L mutant strains. As the mutations can not directly affect protein synthesis, the low levels of HBsAg in cell supernatant and blood serum in rtA181T/sW172* mutant viral strain should be a direct result of the retention of HBsAg in liver cells. In another word, the HBsAg coding by rtA181T/sW172L mutant strain (substituted HBsAg) could be normally secreted or transferred to the outside of the cells, but the HBsAg coding by rtA181T/sW172* mutant strain (truncated HBsAg) could not. As mentioned before, the virion assembly and secretion were inseparable from the participation of HBsAg. So the assembly and secretion of virus particles may be different between HBV rtA181T/sW172* and rtA181T/sW172L mutant strains. For rtA181T/sW172L mutant strains, the emergence of substituted HBsAg may not affect the assembly and secretion of virus particles, which may be similar to wild-type HBsAg. However, for rtA181T/sW172* mutant strains, the truncated HBsAg had significant changes in spatial structure of the protein, and the latter would inevitably impair the assembly and secretion of virus particles from intracellular to extracellular fluids. And this may explain the lower levels of HBV DNA in cell supernatant and blood serum in HBV rtA181T/sW172* mutant strain as compared to that in HBV rtA181T/sW172L mutant and wild-type viral strains. It is worth mentioning that antigens in the liver cells may stimulate prolonged immune response, and the adaptive immune response should be responsible for viral clearance and disease pathogenesis during HBV infection^17^. So, there is a strong need for further researches to promote our understanding on the potential pathogenic role of the retention of truncated HBsAg in hepatic cells.

In our previous studies, we reported that the HBV DNA RI levels of the rtA181T/sW172* mutant strain were always higher and sustained longer as compared to wild-type HBV. But in present study, similar findings were not observed in rtA181T/sW172L mutant strain. In fact, the functions of truncated and substituted HBsAg on the regulation of HBV Cp may explain this confusion, because truncated HBsAg could transactivate HBV Cp, while substituted HBsAg and wild-type HBsAg could not. It is known, the Cp overlaps with the coding sequence of HBx protein, which functions as a transcriptional trans-activator of viral genes and regulates transcription of the 3.5 kb HBV mRNA. The latter not only codes HBcAg, HBeAg, and DNA polymerase, but also involves in viral replication as reverse transcription template (pregenomic RNA)^18^. In fact, the pgRNA level of HBV rtA181T/sW172* was 1.2 time as much as pgRNA level of wild type (data unshown). It was worth to mention that our previous study had reported that a minor increasing of HBV pgRNA could result in a significant increasing of HBV DNA replication levels^18^. Hence, the activity of Cp directly influenced HBV transcription and replication^19^.

Detection of HBsAg is achieved by antibody-based assays targeting the ‘a’ determinant, the highly homologous region within HBsAg, which is also used as the main target of hepatitis B vaccines^20^. Recognition of the ‘a’ determinant by anti-HBs depends on its 3D conformation, which also relies on the amino acid sequence of the regions flanking the ‘a’ determinant^21^. As the sW172L and sW172* mutations of HBsAg gene are not inside the ‘a’ determinant, currently commercially antibody for substituted or truncated HBsAg is effective. In addition, the immunohistochemistry results of HBsAg in liver tissue also supported the effectiveness of conventional antibody for the substituted and truncated HBsAg detection.

It must be noticed that just an *in vitro* experiment is not enough to fully reveal the differences between HBV rtA181T/sW172* and rtA181T/sW172L mutant strains. In this study, there is a little difference in HBV DNA RI of HBV rtA181T/sW172* mutant strain *in vitro* and *in vivo* experiments. And some other *in vitro* experiments also reported a decreased viral replication of HBV rtA181T/sW172*^13^. However, the HBV DNA RI level of HBV rtA181T/sW172* mutant strain *in vivo* was increased. As we mentioned above, the retention of truncated HBsAg in cells may be closely associated with the changes of HBV replication. As the observation time of *in vitro* experiment is relatively short, the amount of truncated HBsAg in cells *in vitro* experiment may be less than that *in vivo* experiment, which may somehow result in the difference of HBV replication between *in vitro* and *in vivo* models. Additionally, the difference in microenvironment and conditions between *in vitro* and *in vivo* models may also affect the levels of viral replication. Thus, to better understand the biological characteristics of HBV mutants, the data from a relevant animal model with HBV replication is also necessary and indispensable.

In summary, this is the first research to study the differences of HBV biological characteristics between rtA181T/sW172* and rtA181T/sW172L mutant strains. Our results have indicated that the appearance of rtA181T/sW172L mutant has no obvious affection to the biological characteristics of HBV; while the appearance of rtA181T/sW172* mutant could cause the retention of HBsAg in hepatic cells, impairment of virus particles secretion from hepatic cells, and enhancement of viral replication in hepatic cells. Based on those significantly differences, when we detected HBV rtA181T mutant in clinical practice, it is necessary and important for us to distinguish the sW172* truncated mutation from the sW172L substituted mutation.

## Materials and Methods

### Study design

This study obtained ethics approval from the Laboratory Animal ethics committee of Sichuan University, and all animal experiments were performed in accordance with relevant guidelines and regulations. This study consisted of two parts. The first part was to compare the DNA replication capacity and viral protein expression among different overlapping S gene mutation (sW172L and sW172*) on rtA181T mutant strain replication and proteins expression *in vitro* and *in vivo* HBV replication systems. To complete this experiment, the pHBV-rtA181T/sW172L and pHBV-rtA181T/sW172* mutant plasmids were generated, and corresponding *in vitro* and *in vivo* models of HBV replication were established.

The second part was to evaluate the effect of substituted, truncated and wild-type HBsAg proteins on HBV gene promoter activity. To complete this experiment, the HBsAg eukaryotic expression plasmids of pcDNA-HBs(sW172L), pcDNA-HBs(sW172*) and pcDNA-HBs(wt) were generated, and HBV promoter report gene plasmids and dual-luciferase reporter assay system were also used.

### Plasmid construction

The wild-type pHBV4.1 is an HBV replication competent plasmid, which contains 1.3 copies of HBV genome (subtype ayw) and capable of process complete replication both *in vitro* and *in vivo*. Thus, it could be used to the study the biological characteristics of HBV replication^18^, and reserved in our laboratory. And it is capable of HBV transcription, replication, and expression both *in vitro* and *in vivo*. The pHBV-rtA181T/sW172L and pHBV-rtA181T/sW172* mutant plasmids were generated by site-directed mutagenesis using wild-type pHBV4.1 as the template. Both two mutant plasmids are capable of HBV transcription and replication. And plasmid pHBV-rtA181T/sW172L could express substituted HBsAg, while pHBV-rtA181T/sW172* could express truncated HBsAg.

A series of HBsAg eukaryotic expression plasmids(including pcDNA-HBs(sW172L), pcDNA-HBs(sW172*) and pcDNA-HBs(wt)) were also generated when the substituted, truncated and wild-type HBsAg coding genes (1176 bp) were respectively cloned into the eukaryotic expression plasmid pcDNA3.0, by using the corresponding pHBV-rtA181T/sW172L, pHBV-rtA181T/sW172* and pHBV4.1 as the template. All these plasmids were finally identified by restrictive enzymes digestion and sequencing.

### The establishment of *in vitro* and *in vivo* models of HBV replication

To construct HBV replication cell model with above mentioned plasmids (including wild-type pHBV4.1, mutant pHBV-rtA181T/sW172L or pHBV-rtA181T/sW172*), the HepG2 cells were used in this study, which was grown in RPMI-1640 medium supplemented with 10% fetal bovine serumat 37 °C in 5% CO_2_. And HepG2 cells were transiently transfected with 10 μg of wild-type or mutant plasmids using commercial X-tremeGENE HP DNA Transfection Reagent (Roche, Germany). And cell supernatant and cells were collected at 3 days after transient transfection.

To construct a mouse model of HBV replication, specific pathogen-free (SPF) level male BALB/c mice (6–8 weeks old), weighing 18–20 g, were purchased from the Laboratory Animal Center at Sichuan University. As we reported previously, mice were injected via the tail vein with 10 μg of HBV plasmid DNA (wild-type or mutant HBV replication plasmids) in 2.0 ml of saline solution within 5–8 seconds (hydrodynamics-based *in vivo* transfection). And mice (at least three mice in each group) were sacrificed after 1, 3, 5, 7, 10 and 15 days of DNA injection, and the serum and liver tissues were collected. The serum was stored at −20 °C for ELISA analysis, and liver tissue was stored in formalin for immunohistochemical staining or stored at −70 °C prior to analysis for HBV DNA replication intermediates. In present study, both *in vitro* and *in vivo* experiments were repeated for three times.

### The detection of HBV antigens and HBV DNA

In this study, HBsAg were quantified using chemiluminescence (Abbott Laboratories, America) while HBeAg were detected by enzyme-linked immunosorbent assay kits (Shanghai Shiye Kehua Company, China) in cell culture supernatants of HepG_2_ cells and in the serum of mice. Absorbance was measured in a microtiter plate reader with dual-wave length measurement (450/645 nm). Additionally, real-time PCR (qPCR) technology was also used to detect the HBV DNA in cell culture supernatants of HepG2 cells and in the serum of mice, using a diagnostic kit (Da An Gene, Guangzhou, China).

The immunohistochemical staining was applied to detect the intrahepatic HBsAg and HBcAg in the formalin-fixed and paraffin-embedded liver tissues according to the manufacturer’s protocol, by using specific antibodies against HBsAg (mouse anti-HBs, Thermol) and HBcAg (rabbit anti-HBc, NEOMARKERS), respectively.

### The detection of viral replication intermediates

For the isolation of viral DNA replication intermediates, the cells were lysed and DNA filter hybridization analyses were performed using 30 μl of viral DNA replication intermediates as described previously. Frozen liver tissues were mechanically pulverized in liquid nitrogen and HBV DNA replication intermediates were isolated from one hundred and twenty micrograms of liver tissue powder as described previously. And these HBV DNA replication intermediates were diluted to 30 μL with TE buffer.

All of viral replication intermediates was analyzed by Southern blotting as previously described. Membranes were hybridized with digoxigenin-labeled (Roche Applied Science) HBV ayw genomic DNA to detect HBV sequences. The levels of viral replication intermediates were calculated by the Quantity One software according to manufacturer’s instructions (Bio-Rad).

### The analysis of HBV promoter activity

Transfections using luciferase reporter gene construct were performed in 6-well plates containing approximately 3 × 10^4^ HepG_2_ cells per mL. The transfected DNA mixture comprised 5 μg of HBV promoter report gene plasmid (CpLUC or PS1pLUC or SpLUC or XpLUC) and 0.5 μg of HBsAg eukaryotic expression plasmid (pcDNA-HBs(wt) or pcDNA-HBs(sW172*) or pcDNA-HBs(sW172L)) or the negative control plasmid pcDNA3.1. Additionally, the plasmid PRL-SV40 was also co-transfected and served as an internal control for transfection efficiency.

Total cell lysates were prepared from cells harvested 40 to 48 h after transfection. Luciferase assay was done using 10 μl of the transfected cell lysates with the dual-luciferase reporter assay system as instructed by the manufacturer. Ratios of the firefly luciferase activities and Renilla luciferase activity were calculated as relative luciferase activities (F/R). The relative luciferase activity levels of the plasmid pcDNA3.1 were defined as 1.

## Additional Information

**How to cite this article**: Zhou, L.-Y. *et al*. Biological characteristics comparison of HBV rtA181T mutants with truncated or substituted HBsAg expression *in vitro* and *in vivo* model systems. *Sci. Rep.* **6**, 39260; doi: 10.1038/srep39260 (2016).

**Publisher’s note:** Springer Nature remains neutral with regard to jurisdictional claims in published maps and institutional affiliations.
